# Using Active Standing Orthostatic Stress Test to Assess Physiological Responses in Individuals with Long COVID: A Systematic Review

**DOI:** 10.3390/jcm14228139

**Published:** 2025-11-17

**Authors:** Faith Olarinde, Albená Nunes-Silva, Diana C. Sanchez-Ramirez, Yannick Molgat-Seon, Rodrigo Villar

**Affiliations:** 1 Cardiorespiratory & Physiology of Exercise Research Laboratory, Faculty of Kinesiology and Recreation Management, University of Manitoba, Winnipeg, MB R3T 2N2, Canada; olarindf@myumanitoba.ca; 2 Inflammation and Exercise Immunology Laboratory (LABIIEX), Physical Education School, Federal University of Ouro Preto, Ouro Preto 35400-000, Brazil; albena.silva@ufop.edu.br; 3 Department of Respiratory Therapy, Rady Faculty of Health Sciences, University of Manitoba, Winnipeg, MB R3E 0T6, Canada; diana.sanchez-ramirez@umanitoba.ca; 4 Department of Kinesiology and Applied Health, University of Winnipeg, Winnipeg, MB R3B 2E9, Canada; y.molgat-seon@uwinnipeg.ca; 5 Department of Physiology and Pathophysiology, University of Manitoba, Winnipeg, MB R3T 2N2, Canada; 6 Centre on Aging, University of Manitoba, Winnipeg, MB R3T 2N2, Canada

**Keywords:** long COVID, orthostatic stress, active standing test, autonomic responses, cardiovascular responses, orthostatic hypotension, orthostatic intolerance

## Abstract

**Background/Objectives**: Individuals experiencing long COVID (LC) frequently report orthostatic intolerance symptoms, which may be linked to autonomic and cardiovascular dysfunction. The active standing test provides a simple, clinically relevant means to assess these impairments. This systematic review aims to determine the use of the active standing orthostatic stress test in evaluating cardiovascular, autonomic, and respiratory responses in people experiencing LC. **Methods**: A systematic search, according to PRISMA guidelines, was conducted in PubMed, MEDLINE, EMBASE, CINAHL, and Scopus for articles published between 2020 and 2025. This study was registered in PROSPERO CRD-42024615872. Studies were included if they used the active standing test, enrolled adults (≥18 years), included both long COVID and healthy control groups, used continuous beat-to-beat measurements, and reported physiological outcomes. Risk of bias was assessed using the nine-point Newcastle–Ottawa Scale. **Results**: Three studies (216 participants experiencing LC and 186 controls) met the inclusion criteria. Across studies, LC individuals consistently exhibited elevated heart rate in both supine and standing positions. However, blood pressure findings were more variable: only one study reported 13% of participants met orthostatic hypotension criteria, while another found significant increases in diastolic blood pressure during standing. Long COVID groups also showed reduced heart rate variability compared to controls. **Conclusions**: Individuals experiencing LC show elevated heart rate and impaired autonomic function during active standing, with subgroup-specific blood pressure changes. These alterations may contribute to dizziness, fatigue, and reduced activity tolerance. Incorporating active standing into clinical assessment could aid early identification of autonomic dysfunction and inform rehabilitation strategies, though more research is urgently needed.

## 1. Introduction

Long COVID, also called post-acute sequelae of COVID-19, post-COVID-19 condition, or chronic COVID syndrome, is when new, ongoing, or worsening symptoms last for three months or more after the severe-acute respiratory syndrome coronavirus infection and cannot be explained by other health problems [1,2]. Common issues include dizziness when standing, fatigue, breathlessness, and “brain fog,” which together can greatly affect daily activities and quality of life [3]. A systematic review reported that nearly 38% of people experienced ongoing fatigue, and more than half had reduced quality of life three to six months after infection [4].

A frequent problem in long COVID (LC) is difficulty remaining upright often due to orthostatic intolerance (OI) [5,6]. OI occurs when the body struggles to adjust to standing by regulating blood flow, leading to dizziness, light-headedness, or even fainting. These symptoms are often linked to postural tachycardia syndrome (POTS) and orthostatic hypotension (OH). POTS is defined as a rise in heart rate of ≥30 bpm within 10 min of standing without a drop in blood pressure (BP). OH is defined as a fall in BP of ≥20 mmHg systolic (SBP) or ≥10 mmHg diastolic (DBP) within three minutes of standing [7,8,9]. Both conditions increase risks such as falls and injuries.

These problems suggest that LC may disrupt multiple body systems, including the autonomic nervous, cardiovascular, and respiratory systems [3,4,10]. To investigate these changes, researchers use orthostatic stress tests such as the active standing test and the head-up tilt test [11,12]. The active standing test is especially important because it mirrors everyday life as people typically perform around 45–60 postural transitions each day [13]. Unlike the tilt test, which passively moves a person upright, active standing engages leg muscles that help blood flow return to the heart, offering a clearer picture of cardiovascular and autonomic responses [12]. The tilt test may also miss very rapid drops in blood pressure within 30 s of standing, known as initial orthostatic hypotension [14,15].

Despite its relevance, studies using the active standing test in people experiencing LC have reported inconsistent results [8,16,17]. For example, Monaghan et al. (2022) found that only 13% of adults with LC met the criteria for POTS, and very few reported symptoms [8]. In contrast, Blitshteyn et al. (2021) reported that 75% of participants showed this response [16]. Studies using the tilt test have also produced highly variable results, as González-Hermosillo et al. (2023), for instance, found increases in BP rather than HR [17]. These inconsistencies may reflect small sample sizes, differences in participants’ age or health, timing after infection, or variations in testing methods (e.g., protocols) [18]. Therefore, this review aims to (i) identify studies that used the active standing test to measure cardiovascular, autonomic, or respiratory responses in people experiencing LC; (ii) describe the physiological measures reported in these studies, and (iii) explore how differences in active standing protocols may explain inconsistent findings.

## 2. Materials and Methods

### 2.1. Search Strategy

The Population, Exposure, Comparison, and Outcome (PECO) framework was used to guide the research question and search strategy [19]. Published literature indexed in PubMed, MEDLINE, EMBASE, CINAHL, and Scopus from January 2020 to August 2024 was initially searched by FO in August 2024. An updated search was performed in May 2025 using the University of Manitoba Libraries platform, with assistance from an experienced librarian (JW). The search strategy included terms related to long COVID (e.g., ‘long COVID’, ‘post COVID’, ‘post-acute sequelae of SARS-CoV-2’, ‘long-haul COVID’), orthostatic stress testing (e.g., ‘orthostatic stress test’, ‘orthostatic hypotension’, ‘postural transition’, ‘hemodynamic responses’), and cardiovascular, autonomic, or respiratory function (e.g., ‘blood pressure’, ‘heart rate’, ‘heart rate variability’, ‘cardiovascular’, ‘autonomic’, and ‘respiratory’). The search was limited to studies published in the English language and from 2020 to 2025. The search strategies are presented in Supplemental Material Table S1. The review was registered with PROSPERO (CRD 42024615872) and followed the Preferred Reporting Items for Systematic Review (PRISMA 2020) guidelines (Supplemental Material PRISMA 2020 Checklist).

### 2.2. Eligibility and Screening Criteria

Studies were included if they (1) reported on cardiovascular, autonomic, or respiratory responses during the active standing test in adults (≥18 years old) experiencing LC; (2) included a healthy control group for comparison; (3) were published between 2020 and 2025; (4) used continuous beat-to-beat measurements of BP or HR; (5) were published in the English language, and were excluded if they did not use an active standing test, only used manual measurements of BP and HR, focused on assessing immunophenotypical biomarkers of LC, used only self-reported surveys or questionnaires (e.g., DePaul symptom questionnaire, COMPASS-32), were published as systematic, scoping, narrative reviews, case studies, qualitative studies, poster or conference abstracts, letters to editors, or editorials.

Title and abstract screening criteria were independently screened by two review authors (FO and AN) using Covidence (www.covidence.org (accessed on 29 August 2024); Extraction v2.0, Melbourne, Australia). Articles identified as potentially eligible by the two review authors were retrieved, and duplicates were either manually (when identified) or automatically (by Covidence) removed. Full texts of potentially eligible studies were screened independently by FO and AN, and RV resolved conflicts when necessary.

### 2.3. Data Extraction

A standardized extraction form was used by two authors (FO and AN) to extract relevant information pertaining to reference data (author, title, journal, year, and country), study characteristics (objective, design, testing setting), and participant characteristics (age, sex, race, number of participants, and health status prior to COVID-19 infection). Study details, including the inclusion and exclusion criteria, proportion of participants who were hospitalized (severity of COVID-19 infection), current medication use, and reported symptoms, were also extracted. The instrumented (devices, active standing protocol, questionnaires), outcome measures (cardiovascular, autonomic, and respiratory variables), the definition of LC used, conclusions and limitations as identified by the study authors, and funding sources were also extracted (Supplementary Material Table S2). No automation tool was used in this process.

### 2.4. Quality and Risk of Bias Assessment

Two reviewers (FO and AN) independently evaluated the quality and risk of bias for each included study using the nine-point Newcastle–Ottawa Quality Assessment Scale (NOS) for case–control studies [20]. Disagreements were resolved by the supervising author (RV). The NOS was tailored to long COVID studies. Studies were categorized based on the NOS score as follows: 0–3 points were considered low quality, 4–6 points moderate quality, and 7–9 points as high quality across the categories of (i) selection, (ii) comparability, and (iii) exposure [20].

Since the NOS was originally designed for cohort and case–control studies, we adapted the case–control scoring tool for long COVID observational cross-sectional studies by modifying some of its domains to better assess exposure and outcome, as described in previous work in the healthcare field [21,22]. Specifically, we interpreted the domain ‘representative of long COVID cases’ as meeting recognized definitions of long COVID and if the sample was recruited from relevant populations (e.g., community, outpatient clinics, tertiary care units, or post-COVID recovery programs), and interpreted ‘exposure’ as encompassing the process by which a study confirmed COVID-19 infection in its participants and whether the statistical test used was appropriate, fully described, or incomplete. A meta-analysis could not be performed due to the small number of studies and heterogeneity across the studies. The modified scoring criteria are provided in Supplemental Material Table S3.

### 2.5. Data Analysis and Synthesis

Descriptive characteristics were used to describe trials and participants. Means, medians, and associated measures of variability (e.g., 95% confidence intervals (CIs), interquartile ranges [IQR]) were applied to continuous variables, while counts and proportions were used for dichotomous and categorical variables. Given the novelty of LC, the anticipated heterogeneity in study designs, and the scarcity of data related to the research question, conducting a meta-analysis was not feasible. Descriptions of individual studies were extracted and presented to show methodological similarities and differences. A narrative synthesis of results was used to summarize and interpret findings.

## 3. Results

### 3.1. Study Selection and Search Strategy

**Figure 1:**
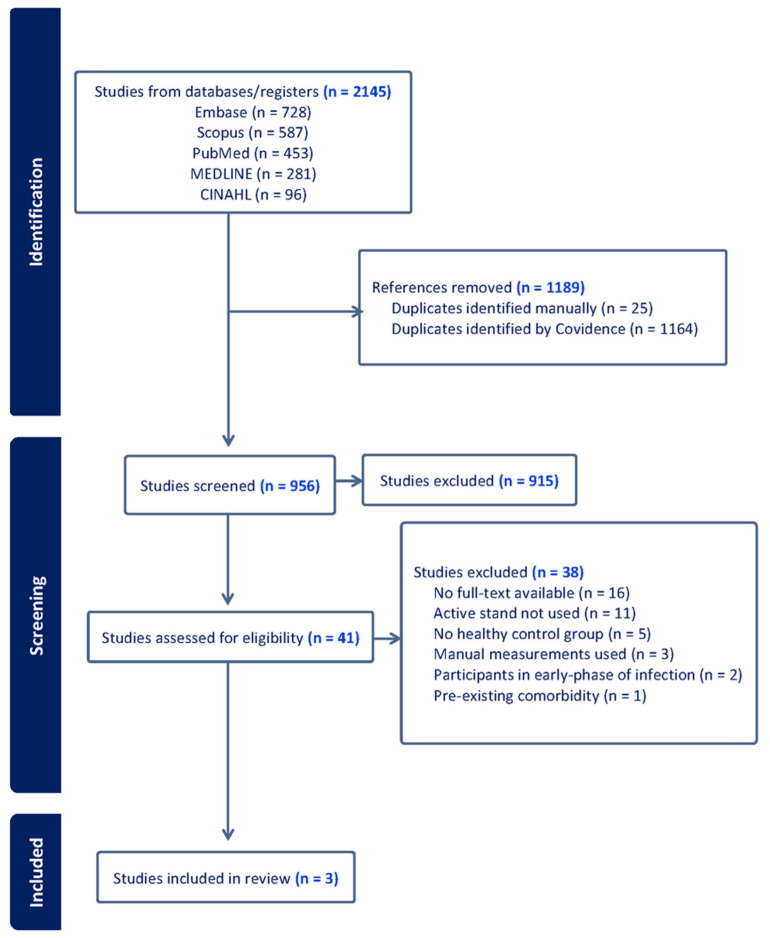
PRISMA flow diagram of study selection.

In total, 2145 references were identified and imported into Covidence for screening through database searches, with 1189 references removed as duplicates. A total of 956 studies were screened by title and abstract following duplicate removal, and 41 studies were screened on the full-text level. Three studies were included in the review following full-text screening. The PRISMA flowchart of study selection is provided in Figure 1.

### 3.2. Study Characteristics

We identified three studies that used the active standing test to evaluate physiological responses in individuals experiencing LC [23,24,25]. These studies included assessments of cardiovascular (e.g., HR, BP), autonomic (e.g., heart rate variability, baroreflex sensitivity) and cardiorespiratory (respiratory sinus arrhythmia) variables. Sample sizes varied across studies, ranging from 30 to 92 LC participants and 33 to 120 healthy control participants.

Methodological approaches differed in terms of data collection protocols (5 min supine and 3 min standing vs. 10 min supine and 10 min standing), and the definition of LC used (WHO’s definition vs. NICE) [23,24,25]. Study design and classification were based on the descriptions provided by the original authors. Study characteristics are presented in Table 1.

### 3.3. Participant Cohort Definitions and Characteristics

Across the included studies, healthy controls were defined as individuals without a history of COVID-19 infection or who had fully recovered from a prior infection [23,24,25]. In Seeley et al. (2025), controls were screened to exclude individuals with frequent syncope, major neurological, cardiac, endocrine, or immune disorders, alcohol or drug dependence, or the use of daily medications other than oral contraceptive pills [24]. Seeley et al. (2025) [24] also included a comparison group of participants with POTS unrelated to long COVID, defined according to international consensus criteria, diagnosed by a physician prior to the COVID-19 pandemic. Hira et al. (2025) [23] also subdivided their long COVID cohort into three categories following initial data analysis—long COVID participants meeting the criteria for initial orthostatic hypotension (LC-IOH), participants with long COVID meeting the criteria for postural orthostatic tachycardia syndrome (LC-POTS), and long COVID participants who did not meet the criteria for any hemodynamic criteria for any autonomic abnormality (LC-none). Vaccination status was not reported by all included studies. Hira et al. (2025) [23] reported that 72% (67/94) of participants were unvaccinated before testing positive for COVID-19, whereas 96% (90/94) were vaccinated at the time of the study test date. However, these values reflect the overall study cohort rather than subgroup-specific (LC vs. healthy controls) distributions. Shah et al. (2022) and Seeley et al. (2025) did not report vaccination status [24,25].

### 3.4. Risk of Bias Assessment

Risk of bias was assessed using the nine-point Newcastle–Ottawa Quality Assessment Scale (NOS) adapted for cross-sectional long COVID studies. All studies were classified as high quality [23,24,25]. The quality of included studies is presented in Supplementary Material Table S4.

### 3.5. Definitions and Diagnostic Criteria

All three studies defined POTS as an increase in HR of ≥30 bpm within 10 min of standing, in the absence of orthostatic hypotension [23,24,25]. Orthostatic hypotension (OH) was defined by Seeley et al. (2025) and Shah et al. (2022) as a ≥20 mmHg drop in SBP or ≥10 mmHg drop in DBP within 3 min of standing [24,25]. Hira et al. (2025) only evaluated initial orthostatic hypotension (IOH), defined as a transient SBP drop of ≥40 mmHg within 15 s of standing, with recovery within 45–60 s, and DBP was not considered in this definition [23].

### 3.6. Heart Rate Responses

All three studies reported elevated HR in LC participants compared to healthy controls in both supine and upright positions [23,24,25]. Supine HR was significantly higher in LC groups, with reported averages ranging from 67 to 88 bpm, compared to 61 to 78 bpm in controls [23,24,25]. The increase in HR following standing was also significantly higher in LC participants, particularly for those meeting the POTS criteria, with standing HR ranging from 99 to 114 bpm compared to 78 to 83 bpm in controls. Subgroup analysis comparisons in Hira et al. (2025) study showed significant differences in supine and standing HR across LC phenotypes, with the LC-POTS group showing the highest increase after standing [23]. Detailed HR responses across studies and subgroups are presented in Table 2.

### 3.7. Blood Pressure Responses

Blood pressure findings were less consistent across studies. Shah et al. (2022) reported that 13% of LC participants (n = 12/92) met the criteria for OH, though absolute values and *p*-values were not reported [25]. Similarly, Seeley et al. (2025) reported no absolute values but found no significant group differences in supine and standing BP (*p* = 0.12) but observed a time-dependent increase in DBP in LC and participants with POTS compared to controls (*p* = 0.03) [24]. In contrast, Hira et al. (2025) [23] reported absolute values and found no overall BP differences between LC and controls. However, subgroup analysis showed higher SBP, DBP, and mean arterial pressure (MAP) in LC participants who met the criteria of initial orthostatic hypotension (LC-IOH) compared to both LC with no abnormalities (LC-none) and LC-POTS groups (all *p* < 0.001). After standing, the LC-IOH showed lower SBP and MAP responses. Additionally, vascular sympathetic modulation, measured by low-frequency systolic blood pressure variability (LF_SBP_), was significantly reduced in LC-none compared to controls during supine and standing positions. Standing LF_SBP_ was also significantly lower in the LC-none group compared to the LC-POTS group. Detailed responses are summarized in Table 3.

### 3.8. Time-Domain Heart Rate Variability Responses

Shah et al. (2022) and Hira et al. (2025) reported decreases in heart rate variability (HRV) in individuals experiencing LC compared to healthy controls [23,25]. Time-domain HRV indices, such as root-mean square of successive R-R interval differences (RMSSD) and standard deviation of R-R intervals (SDNN), were consistently lower in LC participants compared to healthy controls. Shah et al. (2022) [25] also reported a reduction in RMSSD based on COVID-19 severity, finding that the values were lowest in those with severe and moderate illness, with higher values in the mild and asymptomatic groups (*p* < 0.0001). Similarly, subgroup analysis by Hira et al. (2025) [23] also showed significant reductions in heart rate variability indices in the LC-IOH and LC-POTS subgroups compared to the LC-none group during standing. Standing RMSSD was lowest in the LC-POTS group (9.72 [7.37–13] ms) and highest in the LC-none group (19 [16–38] ms), with similar patterns observed for SDNN and pNN50 (percentage of successive R-R intervals differing by more than 50 ms). Detailed time-domain HRV indices across studies and subgroups are presented in Table 4 below.

### 3.9. Frequency-Domain Heart Rate Variability Responses

Frequency-domain HRV indices, including high-frequency (HF), low-frequency (LF), total power (TP), low-frequency to high-frequency ratio (LF/HF), and vagal baroreflex sensitivity (BRSv), were reported only by Hira et al. (2025) [23]. No significant differences were found between LC and control participants during supine. However, after standing, LC participants demonstrated significantly lower HF, LF, and TP compared to controls, with no group differences found in LF/HF ratio or BRSv. Subgroup analysis revealed significant differences in HF, LF, TP, and BRSv across LC phenotypes (LC-IOH, LC-POTS, LC-none) in both supine and standing positions. During supine, the LC-IOH group showed significantly lower HF, LF, and BRSv compared to LC-POTS (all *p* < 0.05), and BRSv was also significantly reduced in LC-IOH compared to LC-none (*p* = 0.01). Following standing, the LC-POTS group had significantly lower HF, LF, and TP compared to LC-none (all *p* < 0.01). BRSv was also significantly lower in both LC-POTS and LC-IOH compared to LC-none. The LF/HF ratio did not significantly differ between groups. Detailed frequency-domain HRV indices across studies and subgroups are presented in Table 5.

### 3.10. Respiratory Responses

Only Seeley et al. (2025) [24] reported respiratory sinus arrhythmia, but no other respiratory system variables were reported. Respiratory sinus arrhythmia was significantly lower in their LC group (9.8 [7.0–14.5] breaths/min) compared to the group with POTS (14.0 [12.1–19.2] breaths/min) and healthy controls (17.4 [12.0–19.5] breaths/min) groups (*p* < 0.001) [24].

## 4. Discussion

This review highlights that the active standing test reveals subtle but clinically relevant cardiovascular and autonomic changes in individuals with long COVID. Across studies, consistent patterns included elevated heart rate (HR) and reduced heart rate variability (HRV) indices (RMSSD, SDNN, LF, HF, TP, BRSv), whereas blood pressure (BP) responses were highly variable [23,24,25]. Notably, the active standing test identified subgroups with exaggerated tachycardic or hypotensive responses, suggesting its value for detecting early autonomic dysfunction and its potential implications for symptom burden, safety, and rehabilitation.

These findings align with growing evidence that autonomic dysfunction is a core feature of long COVID [26,27]. However, the underlying pathophysiology remains speculative. Current research hypothesizes that the exaggerated inflammatory response (or ‘cytokine storm’) triggered by SARS-CoV-2 infection underlies a primary mechanism driving dysautonomia in affected individuals [28].

Across the three included studies [23,24,25], several consistent yet nuanced patterns emerged. Seeley et al. (2025) [24] reported that nearly 80% of LC participants met the diagnostic criteria for POTS. Shah et al. (2022) found a markedly lower incidence (2%) but observed reduced time-domain HRV indices among participants with more severe acute infection, suggesting that severity may influence autonomic response [25]. Hira et al. (2025) [23] further demonstrated heterogeneity within the LC cohort. Some participants showed elevated resting BP and reduced sympathetic vascular modulation followed by exaggerated orthostatic hypotension (LC-IOH group). Others exhibited a pronounced HR increase (LC-POTS group) after standing. These findings suggest that LC-related autonomic dysfunction spans across a spectrum of physiological phenotypes potentially influenced by infection severity, autonomic reactivity, or impaired baroreflex and vascular compensation [3,18,26,27,29]. Similar variability has been reported in other post-viral syndromes [6,7], where mechanisms such as persistent inflammation, microvascular injury, and altered hemodynamics are thought to contribute to orthostatic intolerance [10,30].

These cardiovascular and autonomic alterations have direct implications on quality of life [4,31]. Dysregulation of both sympathetic and parasympathetic function may underline symptoms such as dizziness, fatigue, and exercise intolerance [32,33]. Individuals with LC frequently report substantial limitations in physical and psychosocial functioning, paralleling symptoms seen in other post-viral syndromes such as myalgic encephalomyelitis/chronic fatigue syndrome [4,6]. However, it is suggested that variability in the autonomic and cardiovascular profile of individuals with long COVID might be attributed to factors such as the timing of post-infection autonomic control assessments, age of study participants, disease severity, and general health of individuals prior to SARS-CoV-2 infection [18,34]. Given the multi-system nature of COVID-19, integrating multi-system assessments such as the cerebrovascular, respiratory, and cardiovascular responses within AST protocols could provide a more comprehensive understanding of long COVID’s impact on the physiology of affected individuals. Such an approach would be particularly useful for longitudinal studies assessing recovery trajectories and distinguishing transient dysfunction from persistent dysautonomia and cardiovascular dysregulation. A key challenge identified in this review is methodological heterogeneity across studies. Active standing test protocols differed in duration and timing of measurements, limiting comparability [23,25]. Definitions of long COVID also varied, with some studies relying on self-report while others required a positive polymerase chain reaction or rapid kit confirmation of infection [23,24,25]. Physiological measures were largely restricted to HR and BP, with few assessments of stroke volume, systemic vascular resistance, or cerebral blood flow, which are factors critical for understanding OI pathophysiology [35,36]. These inconsistencies likely explain much of the variability in prevalence estimates and underscore the need for standardized approaches.

This review has several limitations that need to be acknowledged. The number of eligible studies is small, restricting generalizability and precluding meta-analysis. Although our inclusion criteria strengthened rigor by requiring continuous cardiovascular monitoring and the inclusion of healthy controls, this may have excluded relevant but less detailed studies. Publication bias may also favor studies reporting positive findings. Finally, the limited representation of diverse populations reduces the ability to draw conclusions about sex, age, or comorbidity effects. Despite these limitations, this review provides important implications for both research and clinical practice.

Future studies should adopt standardized definitions of long COVID (e.g., National Academies of Sciences, Engineering, and Medicine 2024 consensus definition), consistent active standing test protocols, and beat-to-beat monitoring to capture rapid hemodynamic changes. Expanding beyond heart rate and blood pressure to include stroke volume, systemic vascular resistance, and cerebral blood flow will clarify mechanisms of orthostatic intolerance and guide targeted interventions. Comparative research across post-viral syndromes could also help determine whether these autonomic disturbances are specific to long COVID or reflect broader post-infectious processes.

Ultimately, the active standing test offers a simple and physiologically relevant tool to advance understanding of cardiovascular dysregulation in long COVID, with potential to inform both diagnosis and rehabilitation strategies. Individuals with long COVID show consistent elevations in heart rate and impaired autonomic function during active standing, with varied blood pressure abnormalities. These findings suggest altered autonomic-cardiovascular integration that may contribute to symptoms such as dizziness, fatigue, and reduced tolerance of daily activities. Incorporating active standing into clinical assessment could support earlier identification of autonomic dysfunction, guide rehabilitation strategies, and help tailor management to improve functional recovery in long COVID. The limited evidence base also emphasizes an urgent need for further research evaluating hemodynamic and multisystem responses in this population.

## 5. Conclusions

This systematic review synthesized studies using the active standing test to assess cardiovascular, autonomic, or respiratory responses in long COVID. While limited in number, findings show increased heart rate responses and reduced heart rate variability indices in individuals experiencing long COVID, suggesting persistent autonomic dysfunction. Blood pressure responses were inconsistent, with some subgroup-level differences in systolic and mean arterial pressure. Key gaps included the absence of standardized long COVID definitions, incomplete reporting of absolute values, and the absence of other hemodynamic variables (e.g., cardiac output, stroke volume, systemic vascular resistance) and respiratory measures, limiting comparability. Future research should consider adopting the use of standardized definitions (e.g., National Academies of Sciences, Engineering, and Medicine consensus criteria), using continuous hemodynamic measurement tools, and including broader physiological assessment of variables beyond BP and HR, and incorporate appropriate control groups. A multi-system assessment approach may better characterize dysregulation and inform targeted interventions for long COVID.

## Figures and Tables

**Table 1:** Study characteristics.

Study (Year)	Country	Study Design	Sample Size	Proportion of Females (%)	Age (Mean ± SD, Median [IQR])/[CI] (Years)	Definition of Long COVID	Active Standing Protocol (Timeframe)	Outcome Measures
Shah et al. (2022) [25]	India	Prospective single center	LC = 92 HC = 120	41% 46%	LC = 50.6 ± 12.1 HC = 51.8 ± 4.2	Descriptive definition; no organizational source	5 min supine, 3 min stand	Heart rate variability
Seeley et al. (2025) [24]	Australia	Prospective comparative	LC = 30 POTS = 33 HC = 33	82% 94% 82%	LC = 37 [15] POTS = 28 [14] HC = 28 [23]	WHO’s Delphi Consensus	10 min supine, 10 min standing	Respiratory sinus arrhythmia, heart rate and blood pressure responses
Hira et al. (2025) [23]	Canada	Cross-sectional	LC = 94 HC = 33	81% 76%	LC = 42 [36, 53] HC = 49 [30, 62]	NICE guideline	10 min supine, 10 min standing	Heart rate variability, blood pressure variability, Baroreflex sensitivity, Blood pressure and heart rate responses

Note: SD: standard deviation; IQR: inter-quartile range; CI: confidence interval; LC: long COVID; HC: healthy control; POTS: postural orthostatic tachycardia syndrome; WHO: World Health Organization; NICE: National Institutes for Health and Care Excellence.

**Table 2:** Comparison of heart rate findings across studies.

Study (Year)	Supine HR Findings	Standing HR Findings
Shah et al. (2022) [25]	LC: 88 ± 15 bpm HC: 78 ± 11 bpm *p* = 0.0001	LC-OH: 99 ± 18 bpm No-OH: 86 ± 14 bpm *p* = 0.006 HC: not reported.
Seeley et al. (2025) [24]	LC: 72 ± 13 bpm POTS: 79 ± 12 bpm HC: 68 ± 9 bpm Group (*p* = 0.003) LC *>* HC (*p* = 0.05) LC < POTS *p*: not reported	∆HR (0–10 min) LC: 36 [30–47] bpm POTS: 46 [34–59] bpm HC: 15 [9–20] bpm Group (*p* = 0.001) LC > HC (*p* = 0.001) LC~POTS (*p* = 0.1)
Hira et al. (2025) [23]	LC: 67 [62–75] bpm HC: 61 [56–70] bpm *p* = 0.01 Subgroups: LC-IOH: 67 [61–77] bpm LC-POTS: 68 [65–75] bpm LC-none: 63 [57–73] bpm HC: 61 [56–70] bpm Group (*p* = 0.023) LC-none~HC (*p* = 0.999) LC-none~LC-POTS (*p* = 0.283) LC-none~LC-IOH (*p* = 0.063) LC-POTS~LC-IOH (*p* = 0.999)	LC: 89 [77–106] bpm HC: 78 [69–87] bpm *p* = 0.001 Subgroups: LC-IOH: 85 [77–93] bpm LC-POTS: 114 [103–131] bpm LC-none: 80 [70–88] bpm HC: 78 [69–87] bpm Group (*p* < 0.001) LC-none~HC (*p* = 0.999) LC-none < LC-POTS (*p* = 0.001) LC-none~LC- IOH (*p* = 0.279) LC-POTS > LC-IOH *(p* = 0.001)

Note: OH: orthostatic hypotension; LC: long COVID; HC: healthy controls; LC-OH: long COVID with orthostatic hypotension; no-OH: participants without orthostatic hypotension; POTS: postural orthostatic tachycardia syndrome; HR: heart rate; LC-IOH: participants with long COVID and initial orthostatic hypotension; LC-POTS: participants with long COVID and postural orthostatic tachycardia syndrome; LC-none: participants with long COVID but no abnormalities.

**Table 3:** Comparison of blood pressure response findings across studies.

Variable	Shah et al. (2022) [25]	Seeley et al. (2025) [24]	Hira et al. (2025) [23]
SBP-supine (mmHg)	Absolute values not reported.	No significant group differences (*p* = 0.12). No absolute values reported.	LC: 119 [109–132] mmHg HC: 119 [114–124] mmHg *p* = 0.683. Subgroup: LC-IOH: 130 [123–143] mmHg LC-none: 113 [105–119] mmHg LC-POTS: 112 [103–123] mmHg LC-IOH > LC-none (*p* = 0.001). LC-IOH > LC-POTS (*p* = 0.001).
DBP-supine (mmHg)	Absolute values not reported.	No data reported for DBP in supine.	LC: 71 [64–77] mmHg HC: 67 [64–72] mmHg *p* = 0.065 Subgroup: LC-IOH: 76 [67–79] mmHg LC-none: 67 [63–73] mmHg LC-POTS: 66 [63–73] mmHg LC-IOH > LC-none (*p* = 0.001). LC-IOH > LC-POTS (*p* = 0.009).
MAP-supine (mmHg)	Not reported.	Not reported.	LC: 87 [80–95] mmHg HC: 85 [80–91] mmHg *p* = 0.268 Subgroup: LC-IOH: 94 [87–100] mmHg LC-none: 81 [78–89] mmHg LC-POTS: 81 [77–92] mmHg LC-IOH > LC-none (*p* = 0.001). LC-IOH > LC-POTS (*p* = 0.001).
LF_SBP_ supine (mmHg^2^)	Not reported.	Not reported.	LC: 4.06 [2.69–6.62] mmHg^2^ HC: 5.15 [3.77–9.37] mmHg^2^ LC-none < HC (*p* = 0.001)
SBP-standing (mmHg)	Absolute values not reported. OH identified in 13% of participants.	No significant differences (group*time interaction; *p* = 0.14).	LC vs. Controls: 113 [99–128] vs. 115 [107–132] mmHg; *p* = 0.241. Group main effect: *p* < 0.001. Subgroup: LC-IOH (124 [112–136] > LC-none (109 [95–126]; *p* = 0.031) and LC-POTS (102 [96–117]; *p* < 0.001).
DBP-standing (mmHg)	Absolute values not reported.	DBP increased over time in LC and POTS vs. controls (*p* = 0.03). POTS vs. controls *p* < 0.03; LC vs. control *p* = 0.74.	LC vs. Controls: 72 [64–79] vs. 71 [66–76] mmHg; *p* = 0.999. Group main effect not significant (*p* = 0.135).
MAP-standing (mmHg)	Not reported.	Not reported.	LC vs. Controls: 87 [75–96] vs. 86 [81–97] mmHg; *p* = 0.534. Group main effect *p* = 0.007. Subgroup: LC-IOH (90 [84–100] > LC-none (84 [72–91]; *p* = 0.049) and LC-POTS (79 [74–92]; (*p* = 0.019).
LF_SBP_ standing (mmHg^2^)	Not reported.	Not reported.	LC vs. Controls: 10.5 [6.19–14.8] vs. 14.5 [9.05–30] mmHg^2^; *p* = 0.001. Subgroup: LC-none (6.59 [4.22–12] < controls (14.5 [9.05–30] mmHg^2^; *p* = 0.001) and LC-POTS (10.2 [4.49–17]; *p* = 0.042).

Note. SBP: systolic blood pressure; DBP: diastolic blood pressure; MAP: mean arterial pressure; LF_SBP_: low-frequency systolic blood pressure; LC: long COVID; HC: healthy control; LC-IOH: participants with long COVID and initial orthostatic hypotension; LC-POTS: participants with long COVID and postural orthostatic tachycardia syndrome; LC-none: participants with long COVID but no abnormalities.

**Table 4:** Time-domain heart rate variability indices.

Variable	(Shah et al., 2022) [25]	(Hira et al., 2025) [23]
RMSSD	LC:13.9 ± 11.8 ms HC: 19.9 ± 19.5 ms LC < Controls (*p* = 0.01) Subgroup: Graded reduction by COVID severity (*p* = 0.0001) Asymptomatic: 24.2 ms Mild: 16.3 ms Moderate: 9.3 ms Severe: 7.2 ms	Supine: No significant group-level differences. Standing: LC: 15 [8.9–22] ms HC: 18 [13–32] ms LC < HC (*p* = 0.011). Subgroup: LC-IOH: 15 [9.60–27] ms LC-none: 19 [16–38] ms LC-POTS: 9.72 [7.37–13] ms LC-POTS < LC-none (*p* = 0.001) LC-POTS < LC-IOH (*p* = 0.024)
SDNN	LC: 16.9 ± 12.9 ms HC: 22.5 ± 17.6 ms LC < Controls (*p* = 0.01)	Supine: No significant group-level differences. Standing: LC: 31 [22–43] ms HC: 40 [32–55] ms; LC < HC (*p* = 0.001). Subgroup: LC-none: 37 [28–51] ms LC-POTS: 27 [20–33] ms LC-POTS < LC-none (*p* = 0.018)
pNN50	Not reported	Supine: No significant group-level differences. Standing: LC: 0.23 [0–0.87] ms HC: 0.78 [0.11–2.72] ms LC < HC (*p* = 0.011). Subgroup: LC-IOH: 2.46 [0.32–7.44]% LC-none: 0.83 [0–2.50]% LC-POTS: 8.12 [3.10–13]% LC-POTS > LC-none (*p* = 0.039) LC-POTS > LC-IOH (*p* = 0.026)

Note: LC: long COVID; HC: healthy controls; RMSSD: root-mean square of successive R-R interval differences; SDNN: standard deviation of R-R intervals; pNN50: percentage of successive R-R intervals differing by more than 50 ms. LC-IOH: participants with long COVID and initial orthostatic hypotension; LC-POTS: participants with long COVID and postural orthostatic tachycardia syndrome; LC-none: participants with long COVID but no abnormalities.

**Table 5:** Frequency-domain heart rate variability indices from Hira et al. 2025 [23].

Variable	Supine Findings	Standing Findings
HF	No significant difference between LC and HC. Subgroup: HC: 1136 [262–3121] ms^2^ LC-none: 1919 [725–4921] ms^2^ LC-IOH: 1384 [339–2036] ms^2^ LC-POTS: 2166 [1112–4419] ms^2^ Group (*p* = 0.026) LC-POTS > LC-IOH (*p* = 0.028)	LC: 315 [95–730] ms^2^ HC: 349 [260–1190] ms^2^ LC < HC (*p* = 0.042) Subgroup: HC: 349 [260–1190] ms^2^ LC-none: 578 [337–1881] ms^2^ LC-IOH: 296 [98–871] ms^2^ LC-POTS: 100 [51–339] ms^2^ Group (*p* < 0.001) LC-none > LC-POTS (*p* = 0.001).
LF	No significant differences between LC and HC. Subgroup: HC: 1484 [603–4210] ms^2^ LC-none: 2130 [1142–4393] ms^2^ LC-IOH: 1429 [643–2778] ms^2^ LC-POTS: 2680 [1889–4938] ms^2^ Group (*p* = 0.015) LC-POTS > LC-IOH (*p* = 0.012)	LC: 1203 [483–2212] ms^2^ HC: 1901 [935–4008] ms^2^ LC < HC (*p* = 0.008) Subgroup: HC: 1901 [935–4008] ms^2^ LC-none: 1806 [1106–3932] ms^2^ LC-IOH: 974 [428–1941] ms^2^ LC-POTS: 1080 [298–1671] ms^2^ Group (*p* = 0.001) LC-POTS < LC-none (*p* = 0.020)
TP	No significant differences between LC and HC. Subgroup: HC: 689 [2547–17,839] ms^2^ LC-none: 7702 [4681–14,480] ms^2^ LC-IOH: 5975 [3321–11,206] ms^2^ LC-POTS: 9415 [5096–16,398] ms^2^ Group (*p* = 0.057) LC-POTS > LC-IOH (*p* = 0.030)	LC: 3313 [1773–5559] ms^2^ HC: 5342 [3410–10,550] ms^2^ LC < HC (*p* < 0.001) Subgroup: HC: 5342 [3410–10,550] ms^2^ LC-none: 4938 [301–9406] ms^2^ LC-IOH: 3269 [1757–5130] ms^2^ LC-POTS: 2619 [1413–3949] ms^2^ Group (*p* = 0.001) LC-POTS < LC-none (*p* = 0.006)
BRSv	No significant differences between LC and HC. Subgroup: HC: 6.89 [4.94–11.4] ms/mmHg LC-none: 10.7 [5.35–17.0] ms/mmHg LC-IOH: 6.24 [4.11–10.0] ms/mmHg LC-POTS: 9.81 [8.47–16.0] ms/mmHg Group (*p* = 0.001) LC-POTS > LC-IOH (*p* = 0.010) LC-IOH > LC-none (*p* = 0.001)	No significant differences between LC and controls. Subgroup: Controls: 3.97 [2.93–6.41] ms/mmHg] LC-none: 5.33 [3.34–7.06] ms/mmHg LC-IOH: 2.77 [1.76–5.11] ms/mmHg LC-POTS: 2.85 [1.44–3.93] ms/mmHg Group *p* = 0.001 LC-POTS < LC-none (*p* = 0.001) LC-IOH < LC-none (*p* = 0.005)
LF/HF ratio	No significant differences between groups or subgroups.	No significant differences between groups or subgroups.

Note: LC: long COVID; HC: healthy controls; HF: high-frequency; LF: low-frequency; TP: total power; BRSv: baroreflex sensitivity; LF/HF ratio: low-frequency/high-frequency ratio; LC-IOH: participants with long COVID and initial orthostatic hypotension; LC-POTS: participants with long COVID and postural orthostatic tachycardia syndrome; LC-none: participants with long COVID but no abnormalities.

## Data Availability

No new data were generated or analyzed in support of this research. All data used are available in public databases.

## Supplementary Materials

The following supporting information can be downloaded at https://www.mdpi.com/article/10.3390/jcm14228139/s1, Table S1. Search strategies; Table S2. Variables extracted at full-text level; Table S3. Modified scoring of the Newcastle-Ottawa scale for long-COVID studies; Table S4. Risk of bias assessment; PRISMA 2020 Checklist [37].

## Abbreviations

The following abbreviations are used in this manuscript:

BRSv	Vagal baroreflex sensitivity
CI	Confidence interval
DBP	Diastolic blood pressure
HC	Health controls
HF	High frequency
HR	Heart rate
HR	Heart rate variability
IQR	Inter-quartile range
LC	Long COVID
LC-IOH	Long COVID and initial orthostatic hypotension
LC-None	Long COVID but no abnormalities
LC-POTS	Long COVID and post orthostatic tachycardia syndrome
LF	Lower frequency
LF/HF ratio	Lower to higher frequency ratio
LF_SBP_	Low-frequency systolic blood pressure
MAP	Mean arterial pressure
NASEM	National Academies of Sciences, Engineering, and Medicine
NICE	National Institutes for Health and Care Excellence.
OH	Orthostatic hypotension
pNN50	Percentage of successive R-R intervals differing by more than 50 ms
POTS	Post orthostatic tachycardia syndrome
RMSSD	Root-mean square of successive R-R interval differences
SBP	Systolic blood pressure
SD	Standard deviation
SDNN	Standard deviation of R-R intervals
TP	Total Power
WHO	World Health Organization
