# Supplementary material for: Using Active Standing Orthostatic Stress Test to Assess Physiological Responses in Individuals with Long COVID: A Systematic Review
Source: J Clin Med. 2025 Nov 17;14(22):8139. doi: 10.3390/jcm14228139 (PMC12653395; doi:10.3390/jcm14228139)
Supplement: Supplementary file 1 [file jcm-14-08139-s001.zip › jcm-3974906-supplementary.pdf]

Supplementary Material

Using active standing orthostatic stress test to assess physiological responses in individuals with long-COVID: A systematic review

Supplementary Table S1. Search strategies

Supplementary Table S2. Variables extracted at full-text level

Supplementary Table S3. Modified scoring of the Newcastle-Ottawa scale for long-COVID studies

Supplementary Table S4. Risk of bias assessment

Table S1. Search strategies

MEDLINE Search Strategy

	Search string	Results
1	Middle Aged/ or Aged/ or Adult	20793566
2	Orthostatic intolerance/ or pure autonomic failure/	39447
3	Viral signs/ or blood pressure/ or heart rate/ or respiratory rate/	1251410
4	Posture/ or syncope/ or adult/ or syncope vasovagal/ or hypotension, orthostatic/ or postural orthostatic tachycradia syndrome/ or humans/ or blood pressure	57528966
5	Posture/ or active standing.mp. or Hypotension, orthostatic/	188996
6	Post-acute COVID-19 syndrome/ or long-covid.mp. or SARS-CoV-2/	663440
7	Covid-19/ or post-acute covid-19 syndrome/ or severe acute respiratory syndrome/	1114581
8	Long-haul covid.mp. or Post-acute COVID-19 syndrome/	15612
9	2 and 6	505
10	5 and 6	299
11	4 and 5 and 6 and 7	208

EMBASE Search Strategy

	Search string	Results
1	((long-covid or post-covid* or long-haul covid or PASC) and active standing test).mp.	11
2	(((long-covid or post-covid* or long-haul covid or PASC) and active standing test) or orthostatic stress test or postural transition).mp.	142
3	(((((long-covid or post-covid* or long-haul covid or PASC) and active standing test) or orthostatic stress test or postural transition) and hemodynamic responses) or orthostatic intolerance or orthostatic hypoten*).mp.	28689
4	Limit 4 to yr = “2020 – Current”	5276

CINAHL Search Strategy

	Search string	Results
S9	S1 AND S6 (narrowed by language: English, and publication date: 2020 - 2025)	80
S8	S1 AND S6 (narrowed by age: all adult)	81
S7	S1 AND S6	438
S6	Post-acute sequalae of covid-19 OR PASC OR post-covid syndrome OR long haulers OR chronic COVID AND orthostatic stress test OR orthostatic challenge OR active standing OR tilt table test OR postural stress OR orthostatic intolerance AND cardiovascular response OR heart rate variability OR blood pressure regulation OR autonomic dysfunction OR orthostatic hypotension OR postural tachycardia syndrome	14, 545
S5	S1 and S2 and S4	1
S4	active stand test or orthostatic stress test and long-covid	17
S3	S1 AND S2	6
S2	orthostatic hypotension’ or ‘postural hypotension’ or ‘postural drop	2,673
S1	long covid or chronic covid-19 or post covid or long haul covid or post covid-19	7,114

PUBMED Search Strategy

	Search string	Results
	("long COVID"[Mesh] OR "post-acute sequelae of COVID-19" OR "PASC" OR "post-COVID syndrome" OR "long-haul COVID" OR "long haulers" OR "chronic COVID") AND ("orthostatic stress test" OR "orthostatic challenge" OR "active standing" OR "tilt table test" OR "postural stress" OR "orthostatic intolerance") AND ("cardiovascular response" OR "heart rate variability" OR "blood pressure regulation" OR "autonomic dysfunction" OR "orthostatic hypotension" OR "postural tachycardia syndrome")	14
	((((((("long-COVID"[MeSH Terms] OR "long-COVID"[All Fields] OR "long-COVID"[All Fields] OR "post-acute sequelae of COVID-19"[All Fields] OR "PASC"[All Fields] OR "post-COVID syndrome"[All Fields] OR "long-haul COVID"[All Fields] OR "long haulers"[All Fields] OR "chronic COVID"[All Fields]) OR ("COVID-19"[MeSH Terms] AND "sequelae"[All Fields]))))) AND ((((("orthostatically"[All Fields] OR "orthostatics"[All Fields] OR "orthostatism"[All Fields] OR "standing position"[MeSH Terms] OR ("standing"[All Fields] AND "position"[All Fields]) OR "standing position"[All Fields] OR "orthostatic"[All Fields]) OR "orthostatic stress test"[All Fields] OR "active standing"[All Fields] OR "tilt table test"[All Fields] OR "postural stress"[All Fields] OR "head-up tilt"[All Fields] OR "head-up tilt test"[All Fields])) OR (("hypotension"[MeSH Terms] OR "hypotension"[All Fields] OR "orthostatic hypotension"[All Fields] OR "hypotensive"[All Fields]) OR "postural tachycardia syndrome"[All Fields] OR "POTS"[All Fields]))) AND ((((("cardiovascular response"[All Fields] OR "heart rate variability"[All Fields] OR "blood pressure regulation"[All Fields] OR "autonomic dysfunction"[All Fields]) OR "autonomic control"[All Fields] OR "autonomic regulation"[All Fields])))))	53
	head-up"[All Fields] AND "tilt"[All Fields] AND ("post acute covid 19 syndrome"[MeSH Terms] OR ("post acute"[All Fields] AND "covid 19"[All Fields] AND "syndrome"[All Fields]) OR "post acute covid 19 syndrome"[All Fields] OR ("long"[All Fields] AND "covid"[All Fields]) OR "long covid"[All Fields])	12
	((("blood pressure"[MeSH Terms] OR ("blood"[All Fields] AND "pressure"[All Fields]) OR "blood pressure"[All Fields] OR "blood pressure determination"[MeSH Terms] OR ("blood"[All Fields] AND "pressure"[All Fields] AND "determination"[All Fields]) OR "blood pressure determination"[All Fields] OR "arterial pressure"[MeSH Terms] OR ("arterial"[All Fields] AND "pressure"[All Fields]) OR "arterial pressure"[All Fields] OR ("heart rate"[MeSH Terms] OR ("heart"[All Fields] AND "rate"[All Fields]) OR "heart rate"[All Fields])) AND ("post acute covid 19 syndrome"[MeSH Terms] OR ("post-acute"[All Fields] AND "covid 19"[All Fields] AND "syndrome"[All Fields]) OR "post acute covid 19 syndrome"[All Fields] OR ("long"[All Fields] AND "covid"[All Fields]) OR "long covid"[All Fields])) OR "postcovid-19"[All Fields] OR "post cov*"[All Fields]) AND ("hypotension, orthostatic"[MeSH Terms] OR ("hypotension"[All Fields] AND "orthostatic"[All Fields]) OR "orthostatic hypotension"[All Fields] OR ("orthostatic"[All Fields] AND "hypotension"[All Fields])) AND ("post acute covid 19 syndrome"[MeSH Terms] OR ("post-acute"[All Fields] AND "covid 19"[All Fields] AND "syndrome"[All Fields]) OR "post acute covid 19 syndrome"[All Fields] OR ("long"[All Fields] AND "covid"[All Fields]) OR	2

	"long covid"[All Fields]) AND "long cov*"[All Fields] AND ("post-acute"[All Fields] AND "sequ*"[All Fields])	
	("haemodynamic"[All Fields] OR "hemodynamics"[MeSH Terms] OR "hemodynamics"[All Fields] OR "hemodynamic"[All Fields] OR "haemodynamical"[All Fields] OR "haemodynamically"[All Fields] OR "haemodynamics"[All Fields] OR "hemodynamical"[All Fields] OR "hemodynamically"[All Fields]) AND ("response"[All Fields] OR "responses"[All Fields] OR "responsive"[All Fields] OR "responsiveness"[All Fields] OR "responsivenesses"[All Fields] OR "responsives"[All Fields] OR "responsivities"[All Fields] OR "responsivity"[All Fields]) AND ("post acute covid 19 syndrome"[MeSH Terms] OR ("post acute"[All Fields] AND "covid 19"[All Fields] AND "syndrome"[All Fields]) OR "post acute covid 19 syndrome"[All Fields] OR ("long"[All Fields] AND "covid"[All Fields]) OR "long covid"[All Fields])	59

SCOPUS Search Strategy

	Search string	Results
	(ALL(long AND covid OR pasc OR long-haul AND covid OR chronic AND covid OR post-acute AND seque*) AND TITLE-ABS-KEY(active AND stand* OR orthostatic AND stress OR head AND up AND tilt OR hutt)) AND PUBYEAR > 2019 AND PUBYEAR < 2025	4

Table S2. Variables extracted at full-text level

Reference information	Author(s)<br>Title<br>Journal<br>Year<br>Country
Study information	Study objective<br>Study design<br>Testing setting(s)<br>Number of participants<br>Inclusion and exclusion criteria
Demographic	Age<br>Sex assigned at birth<br>Race<br>Symptoms reported<br>Health status<br>Medication use<br>Hospitalization status during COVID-19 infection
Instrumented measurement information	Techniques/devices used/instruments (protocol, questionnaires)<br>Active standing protocol (timeframe)
Blood pressure and heart rate measurements	Continuous (e.g. using Finometer Finapres)
Outcome measures	Autonomic nervous system variables (Heart rate variability, etc).
	Cardiovascular system variables (Blood pressure, heart rate, cardiac output, etc.)
	Respiratory system variables (spirometry, volume of oxygen consumption and carbon dioxide production, etc.)
Final reports/Other information	Definition of long-COVID used (if applicable)<br>Conclusions<br>Limitations<br>Funding sources/disclosures

Table S3. Modified scoring of the Newcastle-Ottawa scale for long-COVID studies

Category	Original scoring criteria	Modified scoring criteria for long-COVID
**Selection (maximum 4 points)**	Is the case definition adequate? (maximum 1 point)	Did they state their definition of long-COVID?<br>• Yes (1 point)<br>• No, only stated definition of COVID-19 (0 points)<br>• No definition provided (0 points)
	Representativeness of the cases. (maximum 1 point)	Is the sample representative of long-COVID cases?<br>• Yes (1 point)<br>• No (0 points)
	Selection of controls (maximum 1 point)	Is there inclusion of a healthy control group?<br>• Healthy control (1 point)<br>• No control group (0 points)
	Definition of controls (maximum 1 point)	Did they provide characteristics of the control group?<br>• Yes (1 point)<br>• No description of control group characteristics (0 points)
**Comparability (maximum 2 points)**	Comparability of cases and controls, controlling for confounding variables (maximum 2 points)	• The study controlled for the confirmation of long-COVID (the amount of time passed since infection and presentation for symptoms) (1 point)<br>• The study controlled for age and sex of long-COVID to control subjects (age- and sex-matching) (1 point)<br>• Study did not control for any of these factors (0 points)
**Exposure (maximum 3 points)**	Ascertainment of exposure (maximum 1 point)	How did they confirm COVID-19 infection?<br>• Secure record (positive PCR or rapid kit test) (1 point)<br>• Self-reported a probable infection with current long-COVID symptoms (1 point)<br>• No confirmation or description (0 points)
	Same method of ascertainment for cases and controls (maximum 1 point)	Were the same diagnostic criteria used in determining if they had long-COVID used for the cases and the controls?<br>• Yes (1 point)<br>• No or not reported (0 points)
	Non-response rate (maximum 1 point)	Was the statistical test appropriate and absolute outcome measures values fully reported?<br>• Yes (1 point)<br>• No or incompletely described (0 points)

Table S4. Risk of bias assessment

	**Shah et al., (2022)**	**Seeley et al., (2023)**	**Hira et al., (2025)**
Case definition provided	1	1	1
Representativeness of long-COVID cases	1	1	1
Selection of controls	1	1	1
Definition of controls	1	1	1
Comparability of cases – control for time passed since symptom presentation	0	1	1
Comparability of cases – age- & sex- matching	1	0	1
Ascertainment of the exposure	1	1	1
Same method of ascertainment	1	1	1
Statistical test/absolute values reported	0	0	1
**Total score**	**7**	**7**	**9**
**Quality**	**High**	**High**	**High**

PRISMA

PRISMA 2020 Checklist

Section and Topic	Item #	Checklist item	Location where item is reported
**TITLE**			
Title	1	Identify the report as a systematic review.	Title page
**ABSTRACT**			
Abstract	2	See the PRISMA 2020 for Abstracts checklist.	Abstract
**INTRODUCTION**			
Rationale	3	Describe the rationale for the review in the context of existing knowledge.	Main document (Page 1)
Objectives	4	Provide an explicit statement of the objective(s) or question(s) the review addresses.	Main document (Page 2)
**METHODS**			
Eligibility criteria	5	Specify the inclusion and exclusion criteria for the review and how studies were grouped for the syntheses.	Main document (Page 3)
Information sources	6	Specify all databases, registers, websites, organisations, reference lists and other sources searched or consulted to identify studies. Specify the date when each source was last searched or consulted.	Main document (Page 2)
Search strategy	7	Present the full search strategies for all databases, registers and websites, including any filters and limits used.	Supplementary material table 1
Selection process	8	Specify the methods used to decide whether a study met the inclusion criteria of the review, including how many reviewers screened each record and each report retrieved, whether they worked independently, and if applicable, details of automation tools used in the process.	Main document (Page 3)
Data collection process	9	Specify the methods used to collect data from reports, including how many reviewers collected data from each report, whether they worked independently, any processes for obtaining or confirming data from study investigators, and if applicable, details of automation tools used in the process.	Main document (Pages 3 and 4)
Data items	10a	List and define all outcomes for which data were sought. Specify whether all results that were compatible with each outcome domain in each study were sought (e.g. for all measures, time points, analyses), and if not, the methods used to decide which results to collect.	Main document (Pages 3 and 4)
	10b	List and define all other variables for which data were sought (e.g. participant and intervention characteristics, funding sources). Describe any assumptions made about any missing or unclear information.	Main document (Page 4)
Study risk of bias assessment	11	Specify the methods used to assess risk of bias in the included studies, including details of the tool(s) used, how many reviewers assessed each study and whether they worked independently, and if applicable, details of automation tools used in	Main document

Section and Topic	Item #	Checklist item	Location where item is reported
		the process.	(Page 4)
Effect measures	12	Specify for each outcome the effect measure(s) (e.g. risk ratio, mean difference) used in the synthesis or presentation of results.	N/A
Synthesis methods	13a	Describe the processes used to decide which studies were eligible for each synthesis (e.g. tabulating the study intervention characteristics and comparing against the planned groups for each synthesis (item #5)).	Main document (Page 5)
	13b	Describe any methods required to prepare the data for presentation or synthesis, such as handling of missing summary statistics, or data conversions.	Main document (Page 5)
	13c	Describe any methods used to tabulate or visually display results of individual studies and syntheses.	Main document (Page 3-5)
	13d	Describe any methods used to synthesize results and provide a rationale for the choice(s). If meta-analysis was performed, describe the model(s), method(s) to identify the presence and extent of statistical heterogeneity, and software package(s) used.	N/A
	13e	Describe any methods used to explore possible causes of heterogeneity among study results (e.g. subgroup analysis, meta-regression).	N/A
	13f	Describe any sensitivity analyses conducted to assess robustness of the synthesized results.	N/A
Reporting bias assessment	14	Describe any methods used to assess risk of bias due to missing results in a synthesis (arising from reporting biases).	Main document (Page 4)
Certainty assessment	15	Describe any methods used to assess certainty (or confidence) in the body of evidence for an outcome.	N/A
**RESULTS**			
Study selection	16a	Describe the results of the search and selection process, from the number of records identified in the search to the number of studies included in the review, ideally using a flow diagram.	Main document (Page 5)
	16b	Cite studies that might appear to meet the inclusion criteria, but which were excluded, and explain why they were excluded.	Figure 1
Study characteristics	17	Cite each included study and present its characteristics.	Main document (Page 5-6; Table 1)
Risk of bias in studies	18	Present assessments of risk of bias for each included study.	Main document

PRISMA 2020 Checklist

Section and Topic	Item #	Checklist item	Location where item is reported
			(Page 6; Supplementary material table 4)
Results of individual studies	19	For all outcomes, present, for each study: (a) summary statistics for each group (where appropriate) and (b) an effect estimate and its precision (e.g. confidence/credible interval), ideally using structured tables or plots.	Main document (Page 6-9)
Results of syntheses	20a	For each synthesis, briefly summarise the characteristics and risk of bias among contributing studies.	Tables 2-5
	20b	Present results of all statistical syntheses conducted. If meta-analysis was done, present for each the summary estimate and its precision (e.g. confidence/credible interval) and measures of statistical heterogeneity. If comparing groups, describe the direction of the effect.	Main document (Page 6-9; Tables 2-5)
	20c	Present results of all investigations of possible causes of heterogeneity among study results.	N/A
	20d	Present results of all sensitivity analyses conducted to assess the robustness of the synthesized results.	N/A
Reporting biases	21	Present assessments of risk of bias due to missing results (arising from reporting biases) for each synthesis assessed.	Main document (Page 6; supplementary material table 3 and 4)
Certainty of evidence	22	Present assessments of certainty (or confidence) in the body of evidence for each outcome assessed.	N/A
**DISCUSSION**			
Discussion	23a	Provide a general interpretation of the results in the context of other evidence.	Main document (Page 9)
	23b	Discuss any limitations of the evidence included in the review.	Main document (Pages 9-10)
	23c	Discuss any limitations of the review processes used.	Main document (Page 10)
	23d	Discuss implications of the results for practice, policy, and future research.	Main document

PRISMA

PRISMA 2020 Checklist

Section and Topic	Item #	Checklist item	Location where item is reported
			(Page 11)
**OTHER INFORMATION**			
Registration and protocol	24a	Provide registration information for the review, including register name and registration number, or state that the review was not registered.	Main document (Page 3)
	24b	Indicate where the review protocol can be accessed, or state that a protocol was not prepared.	Main document (Page 3)
	24c	Describe and explain any amendments to information provided at registration or in the protocol.	N/A
Support	25	Describe sources of financial or non-financial support for the review, and the role of the funders or sponsors in the review.	Title page (funding sources)
Competing interests	26	Declare any competing interests of review authors.	Title page (conflicting interest)
Availability of data, code and other materials	27	Report which of the following are publicly available and where they can be found: template data collection forms; data extracted from included studies; data used for all analyses; analytic code; any other materials used in the review.	Title page (data availability statement)

From: Page MJ, McKenzie JE, Bossuyt PM, Boutron I, Hoffmann TC, Mulrow CD, et al. The PRISMA 2020 statement: an updated guideline for reporting systematic reviews. BMJ 2021;372:n71. doi: 10.1136/bmj.n71. This work is licensed under CC BY 4.0. To view a copy of this license, visit https://creativecommons.org/licenses/by/4.0/
